# GC-MS-based metabolic profiling of essential oils from *Citrus paradisi*, *Lawsonia inermis*, and *Ruta graveolens* and assessment of their acetylcholinesterase inhibitory potential

**DOI:** 10.3389/fchem.2025.1761973

**Published:** 2026-01-14

**Authors:** Rema M. Amawi, Naglaa S. Ashmawy

**Affiliations:** 1 Mathematics and Sciences Department, Rochester Institute of Technology, Dubai, United Arab Emirates; 2 Department of Pharmaceutical Sciences, College of Pharmacy, Dubai Medical University, Dubai, United Arab Emirates; 3 Department of Pharmacognosy, Faculty of Pharmacy, Ain Shams University, Cairo, Egypt

**Keywords:** Alzheimer’s disease, Citrus paradisi, essential oils, GC-MS, Lawsonia inermis, limonene, phytol, Ruta graveolens

## Abstract

**Introduction:**

Plant-derived essential oils (EOs) are rich sources of bioactive compounds, some of which exhibit acetylcholinesterase (AChE) inhibitory activity and may offer therapeutic potential for the management of Alzheimer’s disease (AD). This study aimed to evaluate the chemical composition and AChE inhibitory potential of essential oils extracted from C*itrus paradisi* (grapefruit), *Lawsonia inermi*s (henna), and *Ruta graveolens* (sadab).

**Methods:**

Essential oils were obtained by hydrodistillation and analyzed using gas chromatography–mass spectrometry (GC–MS) to identify their chemical constituents. AChE inhibitory activity was determined using Ellman’s colorimetric assay, and IC_50_ values were calculated to assess inhibitory potency.

**Results:**

A total of 63 metabolites were identified across the three essential oils, accounting for approximately 90% of their total composition. Grapefruit EO was predominantly composed of limonene (89.94%), henna EO was rich in phytol (41.42%) and limonene (23.02%), while sadab EO was characterized by 1-hexadecanol acetate (26.39%) and phytol (20.54%). Grapefruit EO exhibited the strongest AChE inhibitory activity (IC_50_ = 12.62 ± 0.48 μg/mL), followed by henna EO (IC_50_ = 43.90 ± 0.97 μg/mL), whereas sadab EO showed negligible inhibition.

**Discussion:**

The notable AChE inhibitory activity observed in grapefruit and henna essential oils is likely attributable to their high terpenoid content. These findings suggest that selected plant-derived essential oils may represent promising natural candidates for the prevention or management of neurodegenerative disorders such as Alzheimer’s disease.

## 1 Introduction

Alzheimer’s disease (AD) is a complex, progressive neurodegenerative condition marked by the deterioration of cholinergic neurons. It leads to a significant reduction in acetylcholine (ACh) levels and subsequent cognitive decline, memory impairment, and behavioral disturbances (Chen et al., 2021; Moore et al., 2021). Central to its pathophysiology are neurofibrillary tangles, amyloid plaques, oxidative stress, and heightened acetylcholinesterase (AChE) activity. As AChE is responsible for the hydrolysis of ACh, pharmacological inhibition of this enzyme remains a primary strategy in symptomatic AD treatment. Despite the clinical use of synthetic AChE inhibitors such as donepezil, rivastigmine, and galantamine, their therapeutic efficacy is often compromised by side effects and limited long-term effectiveness (Amawi et al., 2024; Ayaz et al., 2017; Sá et al., 2012).

This limitation has prompted a surge in interest towards plant-based compounds, particularly essential oils (EOs), which are natural, volatile mixtures rich in terpenoids, phenolics, and other bioactive constituents (Arjmand and Dastan, 2020; Dhifi et al., 2016). Their lipophilic nature facilitates passage through the blood-brain barrier, making them viable candidates for central nervous system (CNS) interventions. Numerous studies have demonstrated that EOs from aromatic plants exhibiting notable AChE-inhibitory effects, attributable to constituents (Ashmawy et al., 2024; Dhapola et al., 2024; Hung et al., 2022). Additionally, oxidative stress has been identified as a key contributor to AD pathogenesis, along with other chronic conditions (Dhapola et al., 2024; Juszczyk et al., 2021). This dual role of EOs as antioxidants and anticholinesterase agents enhances their therapeutic appeal in neurodegenerative disease contexts (Konfo et al., 2023; Salim et al., 2025).

Many studies reported the increased use of medicinal plants by population as part of primary healthcare, reflecting their importance in traditional medicine systems and ethnopharmacology (Al-Yateem et al., 2023; Patrício et al., 2022; Tuasha et al., 2023). Moreover, essential oils, beyond medicinal use, are employed in food preservation, cosmetics, and perfumery due to their broad-spectrum bioactivities (Butnariu, 2021; Mounira, 2024). For instance, the genus *Curcuma* is renowned for its phytochemical richness, particularly in curcuminoids, flavonoids, and EOs such as ar-turmerone, caryophyllene oxide, and limonene, which demonstrate antioxidant and neuroprotective properties (Alolga et al., 2022; Peng et al., 2022).

The current study aims to evaluate phytochemical composition and bioactivity, contributing to the discovery of new plant-based neuroprotective agents. Citrus species, particularly *Citrus paradisi* (grapefruit), represent a significant botanical source of bioactive EOs. The *Citrus* genus is lauded for its antioxidant, anti-inflammatory, antimicrobial, and anticholinesterase activities, largely attributed to its rich profile of flavonoids, coumarins, carotenoids, and terpenes such as limonene. These bioactive compounds are concentrated in the fruit peels, making them a valuable by-product in EO production (Saini et al., 2022; Wedamulla et al., 2022). Grapefruit EO has demonstrated notable AChE inhibition, with compounds like nootkatone and auraptene yielding inhibition rates between 17% and 24% at low concentrations (Lim, 2012). Studies suggested grapefruit phenolics enhance antioxidant defences in brain tissue and reduce neuroinflammation *via* suppression of IL-6 and TNF-α cytokines (Deng et al., 2020; Tan and Ismail, 2020).

Beyond neuroprotection, grapefruit EOs have cardioprotective, antihypertensive, antimicrobial, and metabolic regulatory effects (Osaili et al., 2023). *Citrus* EOs are widely used across industries for their antimicrobial, antidiabetic, and insecticidal properties, further underscoring the economic and therapeutic potential of valorizing *Citrus* peel waste, especially in *Citrus*-rich regions (Brah et al., 2023; Reddy et al., 2019).

*Lawsonia inermis*, or henna, is traditionally used for cosmetic purposes but also possesses a rich phytochemical repertoire with medicinal value. Indigenous to Africa and Asia and widely cultivated in tropical regions, henna leaves contain phenolic compounds such as lawsone, gallic acid, quercetin, and tannins, which have been linked to antioxidant, anti-inflammatory, and antimicrobial activities (Elaguel et al., 2019).

Henna EO has also demonstrated hypotensive, analgesic, antipyretic, antibacterial, and immunomodulatory effects. Traditionally, it has been used in treating wounds, gastrointestinal issues, fever, anemia, and tumors (Singh et al., 2015). With only a fraction of the world’s 250,000 to 500,000 plant species having been pharmacologically investigated, henna stands out as a valuable yet underexplored candidate for neuropharmacological applications. This study thus seeks to evaluate the chemical makeup and cholinesterase-inhibitory potential of UAE-derived henna EO, offering insights into its possible role as a neuroprotective agent.

*Ruta graveolens*, known as “sadab” in the UAE, is another aromatic plant of growing interest for neurodegenerative applications. A member of the *Rutaceae* family, sadab is a hardy evergreen shrub historically utilized in Ayurveda, Unani, and homeopathy for its sedative, antispasmodic, emmenagogic, and antimicrobial properties (Ainiwaer et al., 2023; Raghav et al., 2006; Shahrajabian, 2024). The plant is distributed across the Mediterranean, Europe, Africa, and Asia, and has been traditionally employed for treating insomnia, headaches, gastrointestinal disorders, and urinary problems (Ainiwaer et al., 2023).

Recent studies support the broader pharmacological potential of sadab, citing anti-diabetic, antiulcer, anti-inflammatory, and neuroprotective activities (França Orlanda and Nascimento, 2015). Yet, despite earlier phytochemical work in other regions (França Orlanda and Nascimento, 2015; Reddy and Al-Rajab, 2016), UAE-specific studies remain scarce.

Given this context, the present study was designed to extract and characterize essential oils from medicinal plants cultivated in the UAE, specifically grapefruit*,* henna, and sadab. Their cholinesterase-inhibitory activity was evaluated to assess their potential as natural anti-Alzheimer agents. This research not only contributes to the ethnopharmacological validation of traditional remedies but also addresses the urgent global need for effective, safe, and affordable neuroprotective therapeutics. Our study addresses the lack of sufficient research on essential oils in the UAE by providing new insights into the chemical composition and bioactivity of locally available plants. While essential oils have been widely studied in other regions, there is limited scientific data on their pharmacological properties and potential health benefits within the UAE context. By focusing on species commonly found or used in the region, our research contributes valuable evidence to support the safe and effective use of these natural products in local traditional medicine and potential pharmaceutical applications.

## 2 Materials and methods

### 2.1 Plant material

The grapefruit peels, henna leaves, and sadab leaves used in this study were sourced from local markets in Dubai, United Arab Emirates. These plants, long used in traditional herbal medicine, are now partially industrialized and readily available in commercial outlets. Species identification was confirmed through morphological comparison and verification against authenticated reference data to ensure botanical accuracy. A voucher specimen of each plant species has been collected and deposited in the Dubai Medical University Herbarium with the following voucher numbers: DMU-P-CP-101 grapefruit peels, DMU-P-LI-102 henna leaves, and DMU-P-RG-103 sadab leaves.

### 2.2 Extraction of essential oils

During the extraction process, 250 g of the dried plant material were accurately weighed and ground into a fine powder using a mechanical grinder. The resulting powder was transferred into a 5-L round-bottom flask in preparation for distillation. An appropriate volume of distilled water (2 L) was added to the flask, and the essential oil was extracted through hydrodistillation using a Clevenger-type apparatus. The mixture was heated continuously for approximately 4 hours, allowing the volatile components to evaporate and condense for collection.

To enhance the separation and yield of the essential oils from the aqueous layer, diethyl ether was employed following the distillation process. The ether layer containing the essential oils was then subjected to drying using a Termovap sample concentrator and anhydrous sodium sulfate to remove residual moisture, yielding a purified organic extract.

The final essential oil extract was transferred into airtight glass vials and stored at low temperatures (typically 4 C) to preserve its chemical integrity for subsequent analytical and bioactivity assays.

### 2.3 Gas chromatography–mass spectrometry (GC–MS) analysis

The essential oils (EOs) extracted from the three plant species were analyzed through metabolic profiling using gas chromatography–mass spectrometry (GC–MS). The analysis was carried out on a Shimadzu GC/MS-QP 2010 system (Kyoto, Japan), coupled with a Thermo-Finnigan SSQ 7000 quadrupole mass spectrometer (Bremen, Germany). A non-polar DB-5MS fused silica capillary column (30 m × 0.25 mm i. d., 0.25 µm film thickness; Agilent Technologies, Santa Clara, CA, United States) was employed for compound separation. The GC oven was programmed to start at 45 C (held for 120 s), then increase at a rate of 0.08 C/s until reaching 300 C, where it was held constant for 300 s. The injector and detector temperatures were maintained at 250 C and 280 C, respectively. Essential oil samples were diluted to a 1% (v/v) concentration in n-hexane, and 1 µL of each was injected automatically using a 1:15 split ratio. Helium was used as the carrier gas at a constant flow rate of 0.02 mL/s. The mass spectrometer operated in electron impact ionization mode at 70 eV, with the ion source temperature set at 200 C. Mass spectra were collected across a scan range of 35–500 m/z to support compound identification and characterization. Essential oil compositions were identified based on mass spectral data and calculated retention indices (RIs), determined using a homologous series of standard *n*-alkanes (C8–C28) analyzed under identical chromatographic conditions. The *n*-alkane standards, each at a concentration of 1,000 μg/mL in hexane, were obtained from Sigma-Aldrich (St. Louis, MO, United States) and stored at 2 C–8 C. Retention indices for compounds eluting beyond C28 were extrapolated using the linear regression of the retention times of the C8–C28 standard *n*-alkane series. Compound identification was based on mass spectral similarity with entries in the NIST and Wiley libraries and supported by retention index comparison with literature values. Pure analytical standards were not used except for *n*-alkane calibration.

### 2.4 Evaluation of antiacetylcholinesterase activity

Acetylcholinesterase (AChE) activity was evaluated using a modified 96-well microplate assay based on Ellman’s method (Beniaich et al., 2023). This highly sensitive technique measures thiocholine production resulting from the hydrolysis of acetylthiocholine, which continuously reacts with 5,5-dithiobis (2-nitrobenzoic acid) to form a measurable chromophore. Three buffer solutions were prepared as follows: Solution A consisted of 50 mM Tris/HCl, pH 8; Solution B contained 50 mM Tris/HCl, pH 8 with 0.1% bovine serum albumin (fraction V); and Solution C comprised 50 mM Tris/HCl, pH 8 with 0.1 M NaCl and 0.02 M MgCl_2_·6H_2_O. To each well of a 96-well microplate, 25 µL of acetylthiocholine iodide (15 µM), 125 µL of 5,5-dithiobis (2-nitrobenzoic acid) in Solution C (3 µM DTNB or Ellman’s reagent), 50 µL of Solution B, and 25 µL of the test compound - dissolved in methanol (MeOH) and diluted in Solution A to final concentrations of 0.5, 1, 2, 3.9, 7.8, 15.6, … , 500, and 1,000 μg/mL - were added. Each essential oil sample was first dissolved in methanol (MeOH) to prepare a stock solution, then serially diluted in Tris-HCl buffer (Solution A) to obtain final assay concentrations ranging from 0.5 to 1,000 μg/mL. From these, 25 µL was added per well. The final concentrations present in the wells were used to construct dose–response curves and calculate IC_50_ values. At each dilution step, samples were visually inspected to confirm the absence of precipitation or phase separation. Even at the highest concentration (1,000 μg/mL), the EO solutions remained homogeneous when prepared in MeOH and diluted in Tris-HCl buffer and were mixed thoroughly before addition to the wells. The initial absorbance was recorded at 405 nm for 30 s using a Microplate TS-800 reader (BioTek, Winooski, United States). Subsequently, 25 µL of acetylcholinesterase (AChE) enzyme solution (0.22 U/mL) was added to each well, and the absorbance was measured again at 300-s intervals over a total period of 30 min (i.e., six readings per well), during incubation at room temperature (25 C ± 1 C).

IC_50_ values were calculated using non-linear regression based on a four-parameter logistic (4 PL) model. Dose–response curves were generated from three independent experiments, each performed in triplicate, and results are expressed as mean ± standard deviation (SD). The percentage of AChE inhibition was determined by comparing the reaction rates of the test samples to those of the negative control, which consisted of 10% methanol in Solution A and was considered to represent 100% enzymatic activity. Donepezil was used as positive control at concentrations ranging from 0.5 to 1,000 μg/mL. All experiments were conducted in triplicate (n = 3), and results were expressed as mean ± standard deviation.

## 3 Results

### 3.1 Chemical composition of essential oils

Essential oils (EOs) have been successfully extracted by hydrodistillation from grapefruit peel, henna leaves, and sadab leaves. All three EOs were yellow, hydrophobic, and possessed characteristic scents. The essential oil yields obtained *via* hydrodistillation were 1.2% for grapefruit peel, 0.25% for henna leaves, and 0.4% for sadab leaves, calculated relative to the dry weight of the plant material. These yields are in agreement with those reported in previous studies.

**FIGURE 1:**
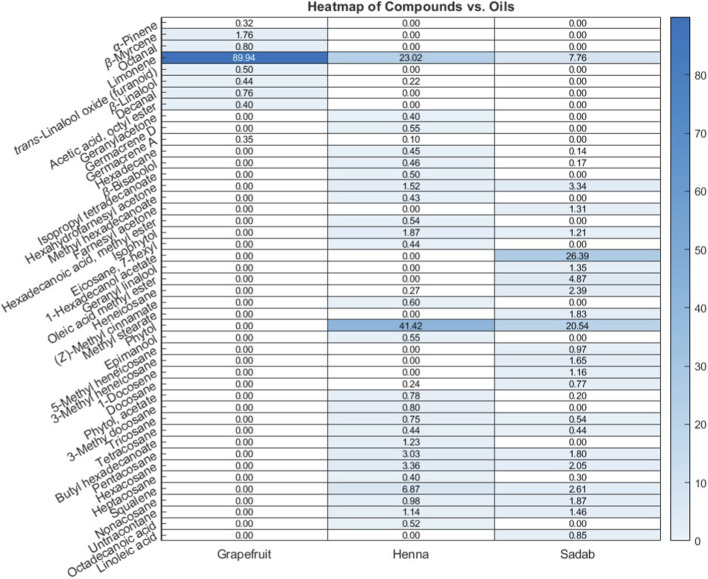
Heatmap illustrating the relative concentrations (%) of identified chemical compounds across three essential oils: grapefruit, henna, and sadab. Rows represent individual volatile and semi-volatile compounds detected by GC–MS analysis while columns correspond to the different essential oil samples. Color intensity (white to dark blue) reflects increasing compound abundance, with numerical values shown within each cell indicating the measured concentration. The heatmap highlights both qualitative and quantitative differences in chemical profiles among the oils, demonstrating distinct compositional patterns as well as compounds uniquely present or enriched in specific essential oils.

Gas chromatography–mass spectrometry (GC-MS) identified a total of 63 compounds across the three EOs (Table 1; Figure 1). In grapefruit EO, limonene was the major component (89.94%), accompanied by minor constituents such as *β*-myrcene, *α-*pinene, and caryophyllene. Henna EO showed a more complex profile, with phytol (41.42%) and limonene (23.02%) as the predominant compounds, along with *β*-bisabolol, hexahydrofarnesyl acetone, and several long-chain hydrocarbons. Sadab EO contained 1-hexadecanol acetate (26.39%), phytol (20.54%), and hexahydrofarnesyl acetone (3.34%) as its primary constituents, along with other fatty acid derivatives and alkanes.

**TABLE 1:** Chemical composition of essential oils based on GC/MS analysis.

No.	Compound	Formula	Molecular structure	KI		Grapefruit EO	Henna EO	Sadab EO
				Cal	Rep			
1	*α-*pinene	C_10_H_16_	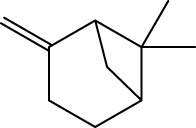	935	935	0.32	-	-
2	*cis-*Sabinene	C_10_H_16_	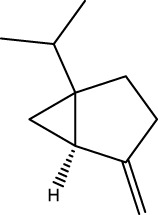	973	974	0.25	-	-
3	*β*-myrcene	C_10_H_16_	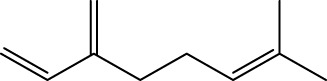	992	992	1.76	-	-
4	Octanal	C_8_H_16_O	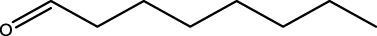	1,004	1,004	0.80	-	-
5	Limonene	C_10_H_16_	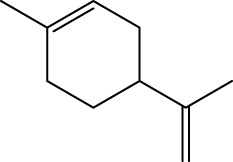	1,032	1,032	89.94	23.02	7.76
6	Linalool oxide	C_10_H_18_O_2_	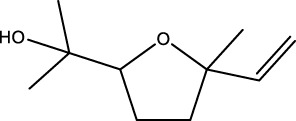	1,073	1,073	-	0.10	-
7	*trans-*Linalool oxide (furanoid)	C_10_H_18_O_2_	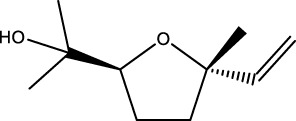	1,073	1,073	0.50	-	-
8	*p-*Cymenene	C_10_H_12_	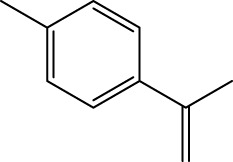	1,089	1,089	0.15	-	-
9	*β*-Linalool	C_10_H_18_O	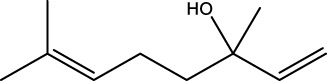	1,102	1,101	0.44	0.22	-
10	Citronellal	C_10_H_18_O	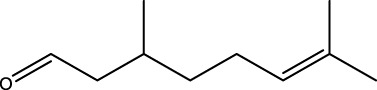	1,155	1,153	0.19	-	-
11	Terpinen-4-ol	C_10_H_18_O	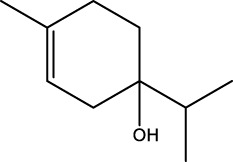	1,180	1,180	0.17	-	-
12	*α-*Terpineol	C_10_H_18_O	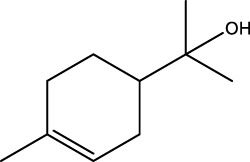	1,193	1,194	0.21	-	-
13	Decanal	C_10_H_20_O	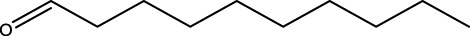	1,207	1,207	0.76	-	-
14	Acetic acid, octyl ester	C_10_H_20_O_2_	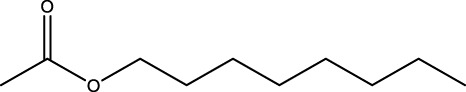	1,214	1,211	0.40	-	-
15	Caryophyllene	C_15_H_24_	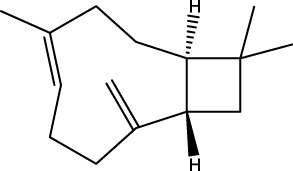	1,425	1,421	0.32	-	-
16	Geranylacetone	C_13_H_22_O	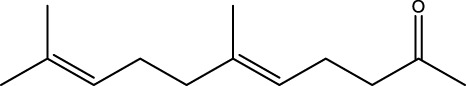	1,458	1,456	-	0.40	-
17	Germacrene D	C_15_H_24_	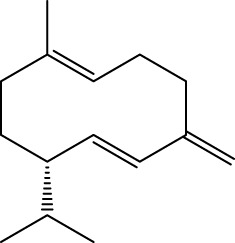	1,494	1,496	-	0.55	-
18	Germacrene A	C_15_H_24_	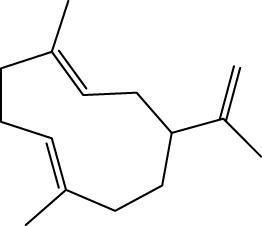	1,502	1,503	0.35	0.10	-
19	(*E*,*Z*)-Pseudoionone	C_13_H_20_O	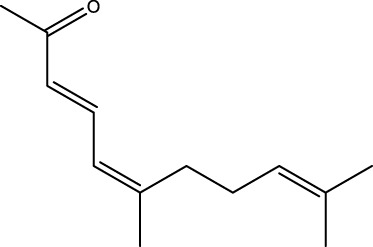	1,534	1,535	-	-	0.09
20	Benzoic acid, hexyl ester	C_13_H_18_O_2_	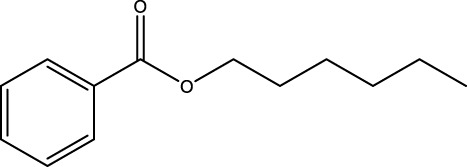	1,579	1,580	-	-	0.18
21	Caryophyllene oxide	C_15_H_24_O	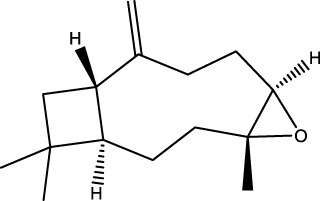	1,592	1,592	-	-	0.27
22	Hexadecane	C_16_H_34_	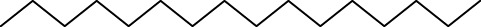	1,600	1,600	-	0.45	0.14
23	*β*-bisabolol	C_15_H_26_O	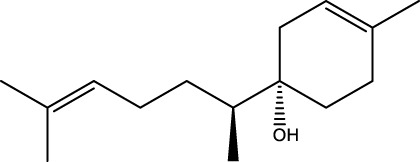	1,670	1,673	-	0.46	0.17
24	*α-*bisabolol	C_15_H_26_O	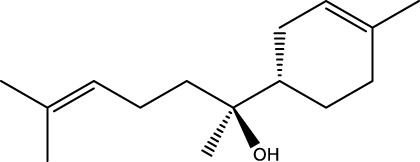	1,671	1,673	0.30	0.26	-
25	Nootkatone	C_15_H_22_O	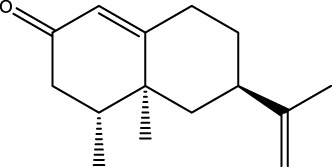	1819	1820	1.44	-	-
26	Isopropyl tetradecanoate	C_17_H_34_O_2_	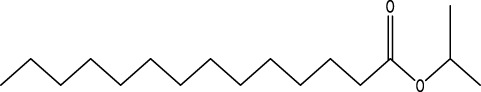	1828	1828	-	0.50	-
27	Neophytadiene	C_20_H_38_	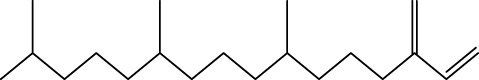	1841	1837	-	0.22	-
28	Hexahydrofarnesyl acetone	C_18_H_36_O	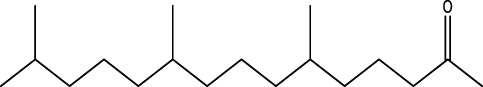	1849	1848	-	1.52	3.34
29	1-Hexadecanol	C_16_H_34_O	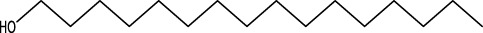	1877	1876	-	-	0.12
30	Nonadecane	C_19_H_40_	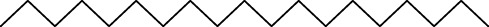	1901	1900	-	-	0.44
31	Methyl hexadecanoate	C_17_H_34_O_2_	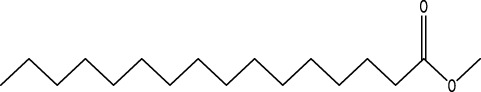	1925	1926	-	0.43	-
32	Farnesyl acetone	C_18_H_30_O	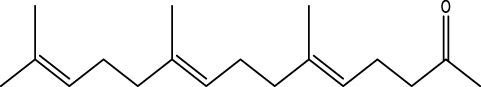	1926	1926	-	-	1.31
33	Hexadecanoic acid, methyl ester	C_17_H_34_O_2_	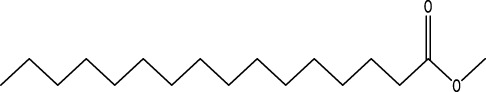	1930	1930	-	0.54	-
34	Isophytol	C_20_H_40_O	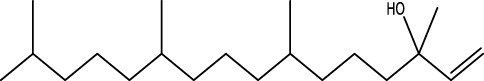	1951	1950	-	1.87	1.21
35	Eicosane, 7-hexyl-	C_26_H_54_	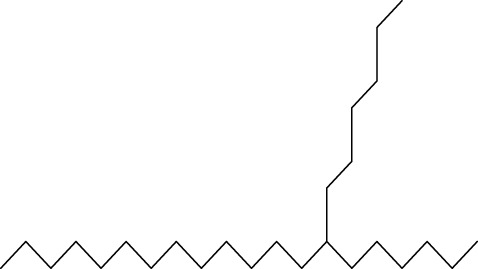	1981	1980	-	0.44	-
36	1-Hexadecanol acetate	C_18_H_36_O_2_	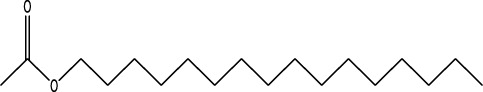	2006	2005	-	-	26.39
37	Geranyl linalool	C_20_H_34_O	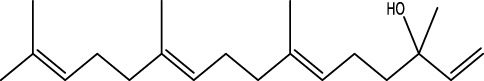	2037	2034	-	-	1.35
38	Oleic acid methyl ester	C_19_H_36_O_2_	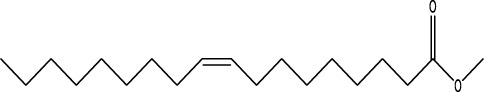	2090	2087	-	-	4.87
39	Heneicosane	C_19_H_34_O_2_	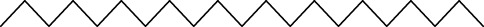	2,100	2,100	-	0.27	2.39
40	(*Z*)-Methyl cinnamate	C_10_H_10_O_2_	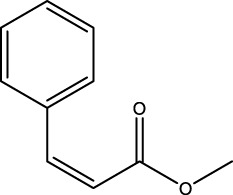	2,107	2,105	-	0.60	-
41	Methyl stearate	C_19_H_38_O_2_	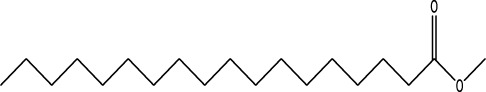	2,109	2,109	-	-	1.83
42	Phytol	C_20_H_40_O	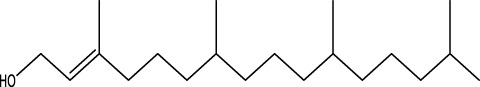	2,122	2,122	-	41.42	20.54
43	Epimanool	C_20_H_34_O	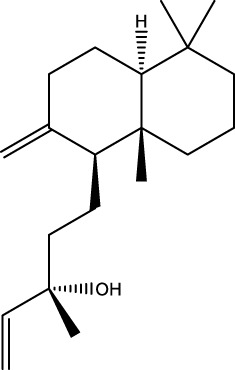	2,133	2,136	-	0.55	-
44	Linoleic acid	C_18_H_32_O_2_	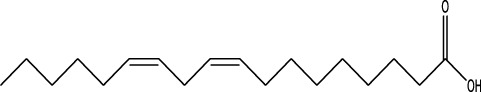	2,143	2,145	-	-	0.85
45	5-Methyl heneicosane	C_22_H_46_	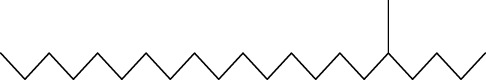	2,150	2,151	-	-	0.97
46	3-Methyl heneicosane	C_22_H_46_	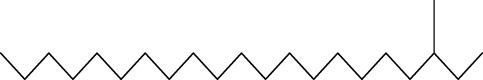	2,173	2,173	-	-	1.65
47	1-Docosene	C_22_H_44_	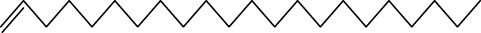	2,193	2,198	-	-	1.16
48	Docosane	C_22_H_46_	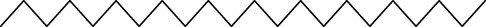	2,200	2,200	-	0.24	0.77
49	5-Ethyl-5-methylnonadecane	C_22_H_46_	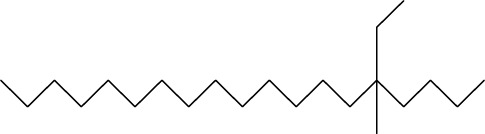	2,213	2,211	-	-	0.23
50	Phytol, acetate	C_22_H_42_O_2_	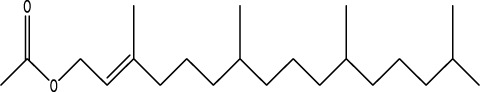	2,223	2,219	-	0.78	0.20
51	3-Methyl docosane	C_23_H_48_	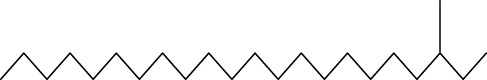	2,278	2,275	-	0.80	-
52	Tricosane	C_23_H_48_	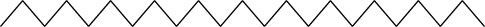	2,302	2,300	-	0.75	0.54
53	2-Methyl-tricosane	C_24_H_50_	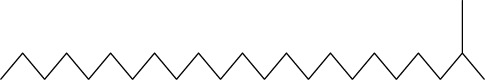	2,363	2,365	-	-	0.38
54	Tetracosane	C_24_H_50_	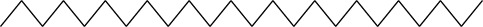	2,399	2,400	-	0.44	0.44
55	Butyl hexadecanoate	C_20_H_40_O_2_	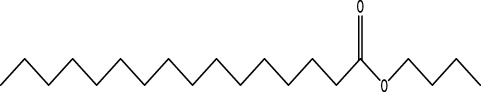	2,420	2,419	-	1.23	-
56	Pentacosane	C_25_H_52_	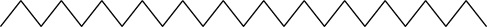	2,502	2,501	-	3.03	1.80
57	Hexacosane	C_26_H_54_	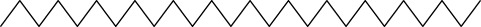	2,599	2,600	-	3.36	2.05
58	Heptacosane	C_27_H_56_	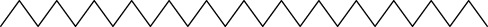	2,701	2,700	-	0.40	0.30
59	Octacosane	C_28_H_58_	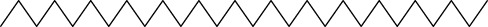	2,799	2,800	-	0.18	0.23
60	Squalene	C_30_H_50_	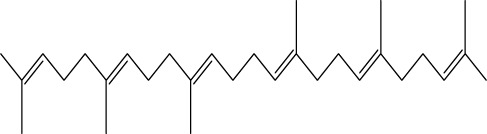	2,836	2,835	-	6.87	2.61
61	Nonacosane	C_29_H_60_	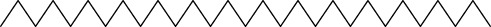	2,901	2,900	-	0.98	1.87
62	Untriacontane	C_31_H_64_	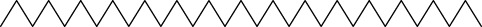	3,100	3,100	-	1.14	1.46
63	Octadecanoic acid	C_18_H_36_O_2_	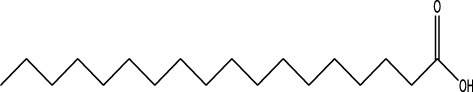	3,104	3,104	-	0.52	-
Total identified compounds %						**98.30**	**94.64**	**89.91**

Compound identification was carried out by comparing their mass spectral data and retention indices with those in the NIST, Mass Spectral Library and the eighth edition of the Wiley Registry of Mass Spectral Data. The percentage content was determined in triplicate using the normalization method based on GC-MS, data. Unidentified in the sample. Standard deviation did not exceed 3% for any of the values. KI, Kovats index calculated on Rtx-5MS, column.

### 3.2 Acetylcholinesterase inhibitory activity

The anti-acetylcholinesterase (AChE) activity of the EOs was assessed *via* Ellman’s colorimetric assay across a concentration gradient (0.5–1,000 μg/mL). Grapefruit EO exhibited the strongest inhibitory effect, with an IC_50_ value of 12.62 ± 0.48 μg/mL. Henna EO showed moderate inhibition (IC_50_ = 43.90 ± 0.97 μg/mL), while sadab EO showed negligible activity, with no measurable IC_50_.

**FIGURE 2:**
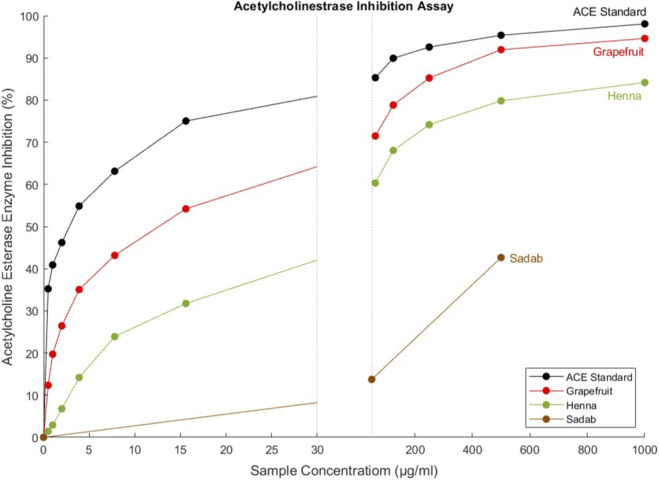
This figure illustrates the acetylcholinesterase (AChE) inhibitory activity of selected plant essential oils (grapefruit, henna, and sadab) compared with a reference AChE inhibitor (ACE standard). Percentage enzyme inhibition is plotted as a function of sample concentration (µg/mL). Data demonstrate a concentration-dependent increase in AChE inhibition for all samples, with the ACE standard showing the highest inhibitory potency across the tested range, followed by grapefruit and henna oils, while sadab exhibits comparatively weaker activity. The figure highlights differential inhibitory profiles among the essential oils, reflecting variations in their bioactive chemical compositions.

Among the active constituents, limonene (89.94% in grapefruit EO) and phytol (41.42% in henna EO) are likely key contributors to the observed inhibition. The dose-dependent inhibition curves are presented in Table 2 and Figure 2. Grapefruit EO’s inhibitory activity closely approached that of the positive control (standard AChE inhibitor), while henna EO displayed partial inhibition across the tested range. No significant inhibition was observed for sadab EO, even at the highest concentrations. Although sadab EO was tested across the full concentration range, measurable inhibition was only detected at 50 μg/mL (13.7%) and 500 μg/mL (42.7%). At intermediate concentrations (62.5–250 μg/mL), inhibition remained negligible and below the sensitivity threshold of the assay, indicating a non-linear and inconsistent response.

**TABLE 2:** Acetylcholinesterase inhibitory activity (%) of plant extracts at various concentrations (µg/mL).

Acetylcholine Esterase Enzyme Inhibition (%)				
Sample conc. (µg/mL)	Grapefruit	Henna	Sadab	ACE Standard
1,000	94.62	84.17	-	98.07
500	91.95	79.83	42.66	95.38
250	85.24	74.16	-	92.57
125	78.82	68.05	-	89.92
62.5	71.48	60.34	-	85.31
50	-	-	13.75	-
31.25	65.09	42.96	-	81.42
15.6	54.21	31.75	-	75.04
7.8	43.17	23.89	-	63.12
3.9	35.06	14.20	-	54.86
2	26.45	6.78	-	46.21
1	19.72	2.91	-	40.89
0.5	12.36	1.43	-	35.23

Dose-dependent AChE inhibitory activity of grapefruit, henna, and sadab extracts compared to the standard inhibitor. Results are expressed as % inhibition based on GC-MS, data, calculated in triplicate.

## 4 Discussion

The findings of this study provide new insights into the chemical and biological properties of essential oils (EOs) extracted from grapefruit peels, henna leaves, and sadab leaves, with a specific focus on their potential as natural acetylcholinesterase (AChE) inhibitors. Among the three oils, grapefruit EO exhibited the strongest anti-AChE activity, as evidenced by its low IC_50_ value (12.62 μg/mL). This potent effect is primarily attributed to its high concentration of limonene, a monoterpene known for its ability to bind the catalytic site of AChE and modulate cholinergic neurotransmission (Eddin et al., 2021; Piccialli et al., 2021). The results align with previous reports on the neuroprotective effects of citrus-derived terpenes, reinforcing the potential of grapefruit EO as a candidate for managing Alzheimer’s disease (AD) symptoms (Matsuzaki and Ohizumi, 2021).

In comparison, henna EO demonstrated moderate but notable AChE inhibitory activity. Although its IC_50_ value was higher than that of grapefruit, the oil still showed biologically meaningful inhibition, likely due to the synergistic actions of phytol and limonene. Both compounds are associated with antioxidant, anti-inflammatory, and neuroprotective properties (Eddin et al., 2023; Sathya et al., 2022). Phytol, in particular, has been implicated in the modulation of oxidative stress and cholinergic signaling pathways relevant to AD pathogenesis (Sharifi-Rad et al., 2022). Moreover, henna EO displayed a chemically diverse profile, suggesting the possibility of multitarget pharmacological effects beyond cholinesterase inhibition. This complexity may offer broader therapeutic potential, especially in multifactorial neurodegenerative conditions.

In contrast, sadab EO exhibited negligible AChE inhibitory activity. Despite the presence of phytol and hexahydrofarnesyl acetone, its chemical profile was dominated by long-chain alkanes and esters, which are generally less reactive and lack the polar functional groups necessary for effective enzyme binding (K et al., 2023; de Oliveira et al., 2019). The poor inhibitory performance of sadab EO supports the view that the presence of phytochemicals alone is not sufficient to guarantee bioactivity. Rather, the structural characteristics of individual constituents, such as molecular size, hydrophobicity, and hydrogen-bonding capacity, play a crucial role in determining their interaction with the AChE active site (de Oliveira et al., 2019; Gyebi et al., 2024; Kamli et al., 2022).

The potent AChE inhibitory activity observed in grapefruit and henna EOs is largely attributed to the high abundance of limonene and phytol, respectively. Although experimental inhibition was quantified, the precise molecular interaction between these compounds and AChE is crucial to understand their pharmacological relevance.

AChE possesses a deep active-site gorge that includes two key regions: the catalytic triad (Ser203, Glu334, His447 in human AChE) and the peripheral anionic site (PAS), which modulates substrate access. Molecular docking studies from previous reports suggest that limonene, a small nonpolar monoterpene, binds primarily through hydrophobic interactions and van der Waals forces within the gorge, potentially near the PAS, altering substrate entry (Kamli et al., 2022).

In contrast, phytol, a larger diterpenoid alcohol, is thought to anchor near the catalytic triad and interact *via* hydrophobic contacts and occasional H-bonding with Trp86 or Tyr337, residues involved in ligand stabilization (Arya et al., 2021; Sathya et al., 2022). Its flexible aliphatic chain allows it to conform to the AChE cavity, partially occluding the active site and thereby reducing substrate hydrolysis.

These mechanistic insights, though derived from existing computational studies, align with the experimental results obtained in this study. Incorporating future molecular docking of UAE-derived limonene and phytol variants could further confirm these interactions and guide structural optimization for enhanced inhibitory activity.

Although sadab EO contains 20.54% phytol, a component that demonstrated moderate AChE inhibition in henna EO, the oil exhibited negligible overall activity. This apparent contradiction may be explained by the chemical context of sadab EO, where active compounds are embedded within a matrix dominated by large, nonpolar, and biologically inert molecules. The high proportion of long-chain esters and saturated hydrocarbons, such as 1-hexadecanol acetate and squalene, likely impedes enzyme access by introducing steric hindrance and reducing solubility or dispersion of the active constituents (de Oliveira et al., 2019; Sathya et al., 2022).

In henna EO, phytol co-exists with limonene (23.02%), a potent AChE inhibitor known to facilitate synergistic or additive effects. In contrast, the absence of such co-activators in sadab may explain its failure to produce a typical sigmoidal dose–response curve. Inhibition was only observed at high concentrations (≥500 μg/mL), and no measurable IC_50_ could be determined, reflecting incomplete and inconsistent inhibition. This suggests that phytol’s bioactivity is not only concentration-dependent but highly sensitive to formulation context, chemical synergy, and delivery environment (de Oliveira et al., 2019; Sathya et al., 2022).

In summary, the comparative analysis highlights grapefruit EO as the most promising cholinesterase inhibitor among the three, followed by henna with moderate potential, while sadab showed minimal activity. These findings contribute to the growing body of evidence supporting the use of EOs as natural AChE inhibitors and underscore the importance of chemical composition and molecular features in predicting bioactivity. Future investigations should focus on isolating and characterizing the most active constituents of the essential oils through bioassay-guided fractionation. Molecular docking and mechanistic studies will help elucidate the interactions between these compounds and the AChE enzyme, while *in vivo* efficacy studies, pharmacokinetic profiling, and safety assessments are critical to validate their therapeutic potential. Additionally, delivery strategies such as encapsulation or nanoformulation may enhance the bioavailability and stability of these components, facilitating their integration into clinical applications.

## 5 Conclusion

This study demonstrated the varying chemical compositions and acetylcholinesterase (AChE) inhibitory activities of essential oils (EOs) derived from grapefruit, henna, and sadab leaves. GC-MS analysis revealed that grapefruit EO was highly enriched in limonene (89.94%), while henna EO contained substantial amounts of phytol (41.42%) and limonene (23.02%). These terpenoid compounds are well-documented for their neuroprotective properties, and their presence likely contributes to the observed bioactivity.

The biological assays confirmed that grapefruit EO exhibited the most potent AChE inhibition, with an IC_50_ value of 12.62 ± 0.48 μg/mL, suggesting high potential as a natural source of cholinesterase-inhibiting agents. Henna EO also demonstrated moderate AChE inhibitory activity (IC_50_ = 43.90 ± 0.97 μg/mL), further supporting the therapeutic potential of plant-derived compounds in neurodegenerative disease contexts. Conversely, sadab EO showed negligible AChE inhibition despite containing compounds such as phytol and hexahydrofarnesyl acetone, highlighting the importance of both concentration and molecular structure in determining enzyme inhibition efficacy.

These results provide promising evidence for the development of natural anti-AChE agents derived from grapefruit and henna essential oils. Given the growing interest in alternative and complementary therapies for Alzheimer’s disease, these oils offer a multifaceted pharmacological profile that may extend beyond enzyme inhibition to include anti-inflammatory and antioxidant effects.

Further investigations should focus on isolating and characterizing the most active constituents through bioassay-guided fractionation. In addition, molecular docking and mechanistic studies would clarify the interactions between these compounds and the AChE enzyme. *In vivo* studies, pharmacokinetics, and safety profiling will be critical next steps to validate their suitability for therapeutic development. Delivery approaches such as nanoencapsulation may also enhance the bioavailability and stability of these essential oil components. Future studies should also explore the antioxidant and anti-inflammatory properties of these essential oils, which are often mechanistically linked to neuroprotection and may further support their use as multi-target agents in Alzheimer’s disease management.

In summary, grapefruit and henna essential oils represent promising plant-based candidates for the development of neuroprotective agents aimed at mitigating the progression or symptoms of Alzheimer’s disease.

## Data Availability

The original contributions presented in the study are included in the article/supplementary material, further inquiries can be directed to the corresponding authors.
